# 

**Published:** 1916-07-22

**Authors:** 


### CONTENTS.

| | PAGE |
|---|---|
| **The War Charities Committee** ... ... ... | 379 |
| **Some Encouraging Experiences** ... ... | 380 |
| **Hospital and Institutional News** ... ... ... | 381 |
| Death of Sir Victor Horsley—The Death of Professor Metchnikoff—Staff Vacancies at London Hospitals—A Central Bureau for Entertainments—Diversifying a Red Cross Week—Nottingham's Claims on Retford's Pockets—The Family Condition of Interned Aliens—Incidents of Ambulance Work: Pelting the Wounded—Board-Room Portraits—A Large Estate for Sheffield Charities—Private Opposition to Public Economy—The Treatment of Venereal Diseases—Economy in the Annual Report—A Hospital for the R.F.C.—A New Isolation Hospital at Bexhill—The Leasowe Open-Air Hospital—The Beckett Denison Infirmary, Doncaster—Progress at the Middleton Sanatorium—A New Open-Air Institution at Manchester—The Treatment of Ophthalmia at Liverpool—The St. Pancras School for Mothers—Consumption in Cottage Homes—Allotments and the Public Health—The Sale of Medicated Wines—The Royal Midland Institution for the Blind, Nottingham—A Man of Many Parts—This Week's Drug Market. | |
| **Post-Mortem Cæsarean Section** ... ... | 387 |
| A Question of Law for Institutional Officers. | |
| **The Equipment of Research Laboratories** ... | 388 |
| Some Applications of Gas Heating. | |
| **Human Temperaments** ... ... ... ... | 389 |
| VII.—The Religious Temperament. | |
| **A Valuable Committee** ... ... ... ... ... | 390 |
| **Sir Perceval A. Nairne** ... ... ... ... | 391 |
| Our Sailors and their Benefactors. | |
| **The Matrons' and Sisters' Department** ... | 393 |
| War, Warriors, and Nurses. | |
| In a French Hospital—Spirituality and the Soldier. | |
| Round the Hospitals. | |
| The Registration Bill—Wise and Foolish Virgins—Nurses and Pathology—Women Workers and the Matrons. | |
| **The Editor's Letter-Box** ... ... ... ... | 395 |
| The Hospitals and Women Students—The Twilight Sleep Propaganda. | |
| **Hospital Results in 1915** ... ... ... ... | 395 |
| **Parliament and the Profession** ... ... ... | 396 |
| **London County Council** ... ... ... ... | 396 |
| **Australian Notes** ... ... ... ... ... ... | 397 |
| **The Drug Resources of the Colonies** ... ... | 398 |
| **Passing Events and Topics** ... ... ... ... | 398 |
| **Matrons' and Sisters' Appointments** ... ... | 398 |
| **Personalia** ... ... ... ... ... ... ... | 398 |
| **Gifts to Hospitals** ... ... ... ... ... ... | 398 |
| **The Institutional Worker** .. .. (Suppl.) | 1 |
| Should Hospital "Letters" be Issued? Answer to the June Question. | |
| Hospitals and Advertising. | |
| A Wood-chopping Brigade. | |
| Enquiries and Answers. | |

